# Neglected hip dislocation: an unusual presentation, its management and review of the literature

**DOI:** 10.1007/s11751-017-0285-7

**Published:** 2017-04-26

**Authors:** Sandeep Kumar, Anshul Dahuja, M. S. Narula, Sangam Garg, Rashmeet Kaur

**Affiliations:** Gian Sagar Medical College Chandigarh Punjab, Patiala, India

**Keywords:** Constrained, Dislocation, Total hip replacement

## Abstract

Neglected traumatic posterior hip dislocation is not an uncommon condition seen in developing countries. Its treatment is fraught with great difficulties and controversies. Here we present a 31-year-old female patient presented to our hospital 2 years after sustaining an accident. She had gone through multiple treatments for her dislocated hip without any success. After arrival to our hospital, patient managed by constrained total hip replacement (THR). Her Harris hip score improved from 48 (preoperative) to 81 (postoperative). On follow-up she had a good range of movement of the hip with no pain, deformity and limb length discrepancy. Constrained THR provides promising results in neglected hip dislocation.

## Introduction

 Neglected traumatic dislocations of the hip are rare in adults. However, in developing countries, unreduced traumatic dislocations are not uncommon. They are usually the result of a motor vehicle accident, often combined with multiple trauma including head injury, fracture of the ipsilateral femur or bilateral injuries, which may detract attention from the dislocation [1]. Neglected hip dislocations occur in situations when the patient does not or cannot seek adequate medical care. As such, chronic dislocations may be observed in patients with a high pain tolerance, patients with decreased cognitive ability to recognize or verbalize their pain and patients with additional injuries that are more obvious or life-threatening [2].

In developing countries, patients usually attend hospital many days after trauma, having often received alternate therapy before. Treatment of neglected dislocation of the hip becomes more difficult to manage as time progresses. The acetabulum becomes filled with fibrous tissue in unreduced dislocations, making reduction impossible by closed means [1].

Reduction of neglected dislocation of hip is not only difficult but results in avascular necrosis and arthritis. Total hip replacement (THR) is recommended for hip dislocations with duration more than 3 months [2].

A complete understanding of the factors that play a role in the etiology of instability of hip and a clear knowledge of treatment options are mandatory for the surgeon tackling this injury. Constrained THR is a viable option for neglected dislocation hip and an unstable hip [3]. The main indications for this technique are instability in the presence of cognitive or neuromuscular disorder and severe abductor deficiency, multiple failed revisions for instability without a constrained socket and an unidentifiable cause of hip instability [3].

## Case report

**Fig. 1:**
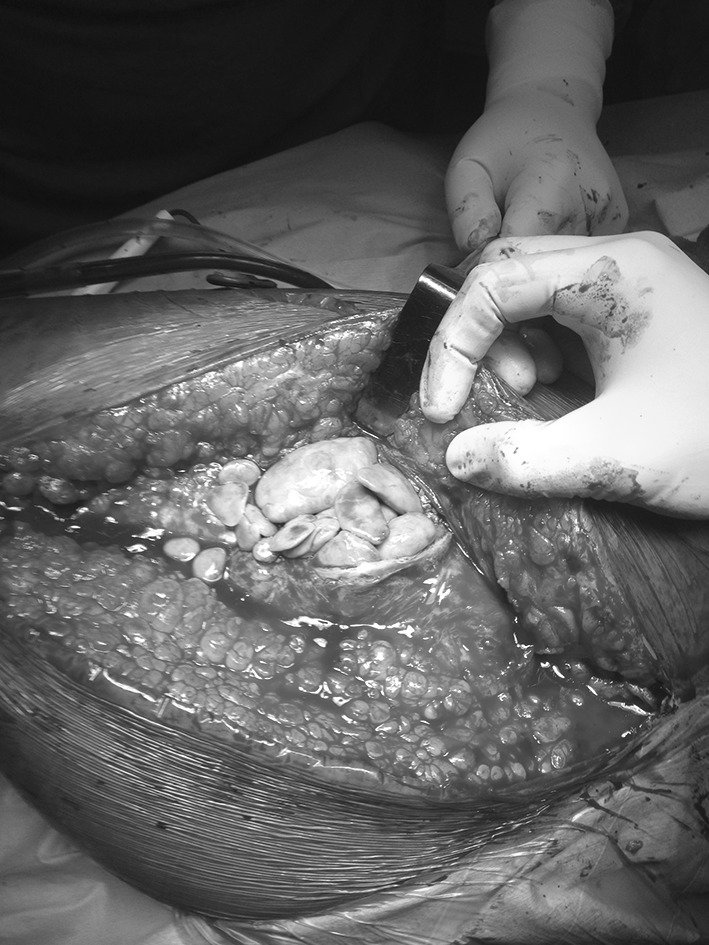
Pebbles of necrotic muscle

**Fig. 2:**
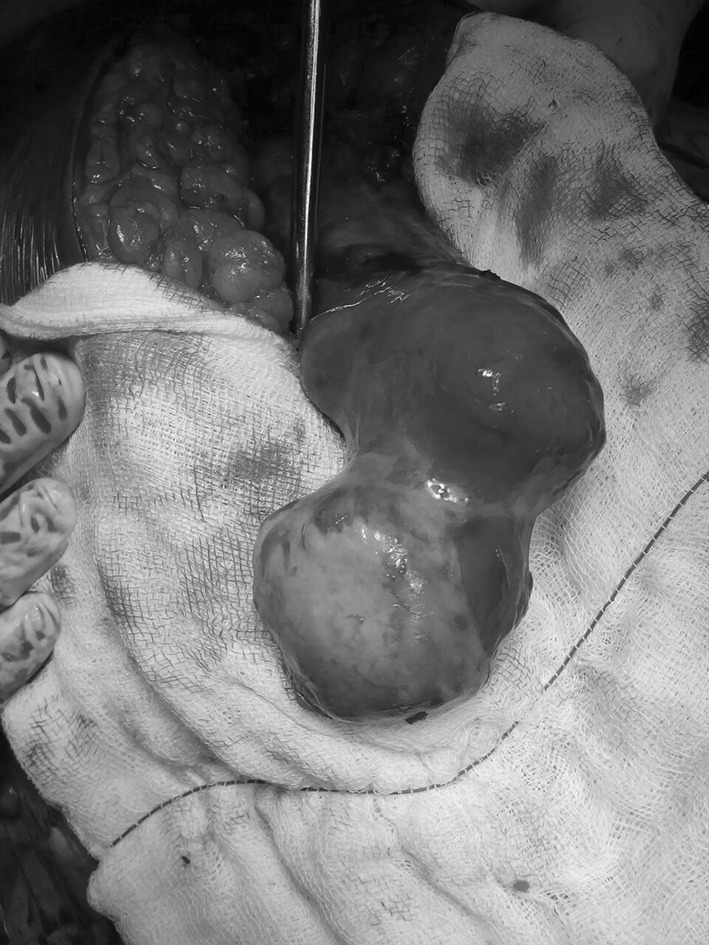
Proximal femur devoid of all muscular attachments

**Fig. 3:**
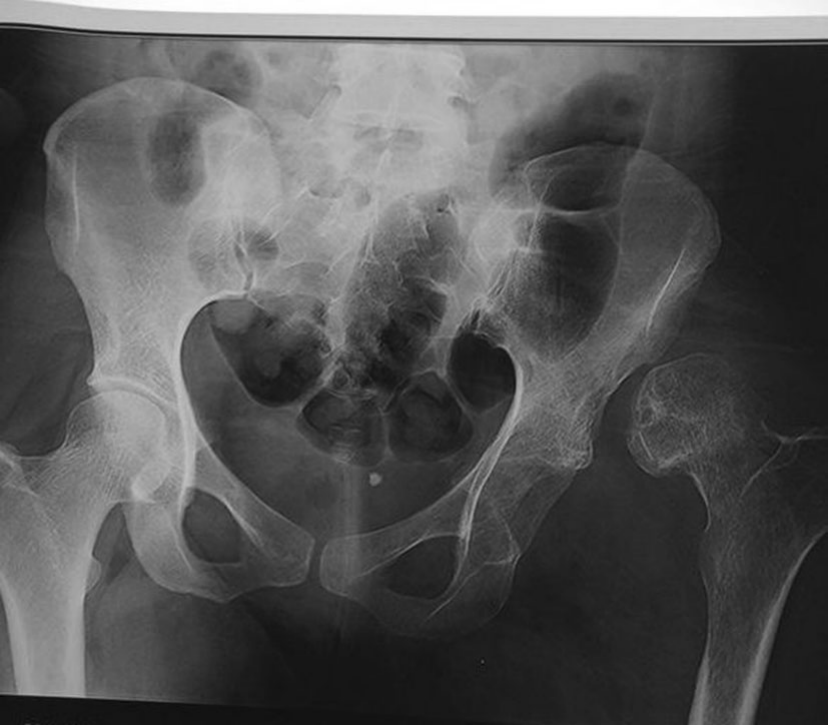
Preoperative X-ray of pelvis with neglected hip dislocation with false hip joint

**Fig. 4:**
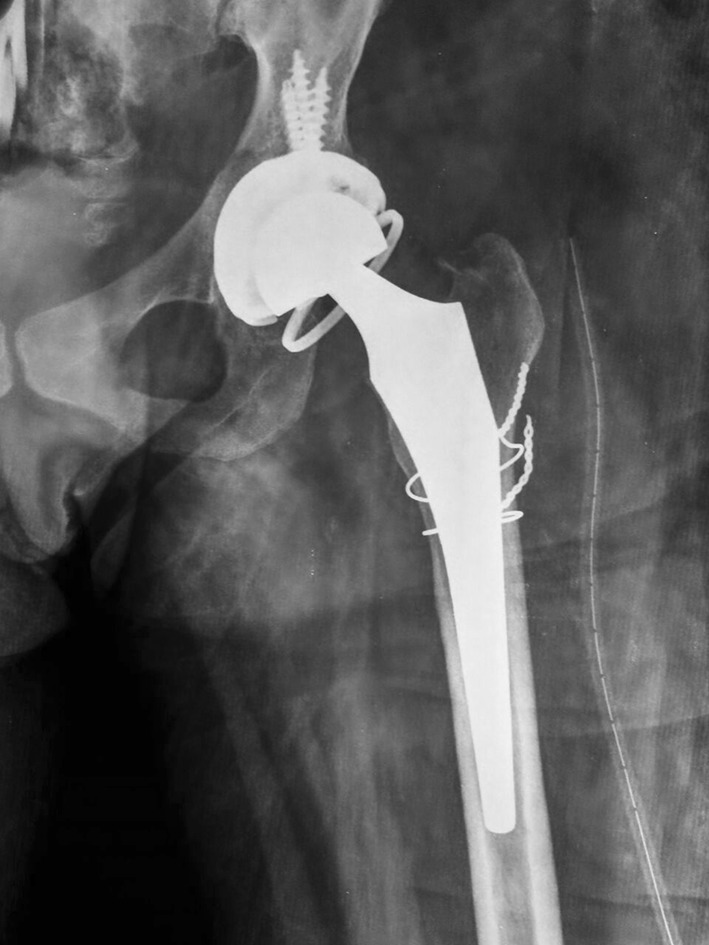
Postoperative X-ray of the patient with total hip replacement with constrained acetabular component

A 31-year-old female sustained a fall while climbing a mountain, resulting in an injury of her left hip, causing pain and an inability to weight bear. She saw a local doctor, who diagnosed a dislocation of her hip and attempted a reduction, which failed, as the hip remained painful und unstable. Six months after the injury, the patient consulted an orthopedic surgeon, who attempted an open reduction of the hip, but he was unable to reduce the hip and abandoned treatment (Figs. 1, 2, 3, 4).

Eighteen months after the initial injury, the patient saw another orthopedic surgeon, who attempted a closed reduction with skeletal traction. The hip remained unreduced despite 2 months of continued traction, and the treatment was discontinued due to pin track infection of the skeletal traction pin.

We saw this patient 2 years after the initial injury complaining of hip pain. She was walking with an antalgic gait and had a decreased range of motion of her hip. The limb was in fixed adduction and internal rotation. She had a leg length discrepancy due to shortening of 6 cm. Her hip movements were restricted and painful. Radiographs confirmed a persistent dislocation of the hip, with a false acetabulum in the left supra-acetabular region.

Initially a total hip replacement combined with a sub-trochanteric osteotomy was planned for this patient with 2 weeks prior skeletal traction. During surgery, we encountered necrotic soft tissues, with the proximal end of the femur devoid of all muscular attachments. A debridement of all necrotic tissue was performed, and tissue was sent for histopathology, which did not isolate any microorganisms. A delayed THR with a constrained acetabular insert for stability was performed a week later. The leg lengths were equalized, and the postoperative recovery was uneventful. Roentgenograms showed satisfactory position and fitting of the prosthesis. Her physiotherapy was started soon postoperatively, and she was discharged on the twelfth postoperative day. Her functional status improved on every successive follow-up visit, and the HHS score improved from 48 (preoperatively) to 81 (postoperatively). She had no pain, a mild residual Trendelenburg gait due to her abductor insufficiency and excellent function.

## Discussion

The ongoing discussion of the best treatment option for the management of neglected dislocation hip continues and remains controversial. Various studies reported different methods for operative treatment of neglected fracture–dislocations including the use of a sub-trochanteric osteotomy, the Girdlestone procedure, hip arthrodesis, hemiarthroplasty and total hip replacement [4]. All these procedures have their merits and give different outcomes. The results can be further altered by avascular necrosis of the femoral head which occurs in more than 50% of these cases. Garrett et al. [2] has recommended total hip arthroplasty for hips with posterior dislocations classified as type IV (fracture of the acetabular rim and floor) or type V (fracture of the femoral head with or without other fractures) with dislocations for more than 3 months.

According to some studies, closed reduction and manipulation under general anesthesia is possible if dislocation is of a relatively short duration (2–4 weeks) [4, 5]. Closed reduction and skeletal traction with the limb in abduction had some good results in selected cases [4], using skeletal traction (15–20% of the patient’s body weight) for 3–5 days, with serial radiograph taken on alternate days. Once the femoral head is at the level of the acetabulum, the limb is then gradually abducted to achieve reduction.

Banskota et al. [4] in their study of eight cases with neglected posterior dislocation on hip reported good results in three cases treating the hip with an open reduction. They achieved a mean Harris hip score of 89 (range 84–96). Leg lengths were within 2 cm in seven of the eight cases, and one patient had a discrepancy greater than 2 cm.

Pai and Kumar [5] in their study of eight patients with neglected posterior dislocation concluded that 66% of patients with dislocation less than 1 year old can be reduced by traction and abduction. They had one patient who had a dislocation older than 1 year with a poor result after reduction. We were not able to achieve reduction by closed means.

Verma et al. [6] in his study of 14 cases neglected dislocation ranging from 14 days to 1 year duration performed open reduction and reported excellent results in four cases, but also had poor outcomes in five cases.

Alternatives to open reduction would be treatment by a sub-trochanteric osteotomy, excision arthroplasty, arthrodesis, hemiarthroplasty and total hip replacement.

Kumar and Jain [7] in their study of 18 patients treated these by open reduction after skeletal traction was unsuccessful. Despite varying degrees of avascular necrosis, they reported excellent results in 17 patients.

Ilyas et al. [8] had reported functional outcomes after THR for neglected fracture–dislocation of the hip in their series.

In a study conducted by Berend et al. [9] using a constrained THR, ten percent were primary hip replacements in patients with an abductor dysfunction secondary to trauma or neuromuscular disease. They reported promising results, which would indicate that constrained THR to be a viable alternate option with abductor dysfunction with a high dislocation risk [9].

Constrained acetabular components use a locking mechanism to capture the femoral head and thereby prevent dislocation. These devices have been used to treat, or in some cases prevent, instability at the cost of decreased range of motion as compared to conventional THR system. The results confirm that the constrained liner is effective in the difficult population with hip instability [10]. However, complications related to the constraining mechanism and increased constraint are of concern. The potential risks associated with constrained acetabular components include component failure, dislocation and increased interfacial stresses resulting in acetabular loosening. Indications for this type of component are limited to patients whose risk of recurrent dislocation or additional surgery exceeds the inherent risks associated with a constrained component [10].

Goetz et al. evaluated 56 hips, with an average follow-up of 10 years, treated with a constrained component. Recurrent dislocation occurred in only 7% of cases [11].

Our case report concurs with these studies favoring a constrained total hip replacement (THR) in an old hip dislocation with compromised soft tissues and doubtful potential stability.

Despite gaining 5–6 cm of limb length, sciatic nerve was unaffected which is indirectly favouring Eggli et al. [12] who found no correlation between the amount of lengthening and sciatic nerve palsy in a large study on THR.

We have not found any other case report with a 2-year-old neglected dislocated hip with complete atrophy of the stabilizing muscles, as in our case, having achieved a satisfactory conclusion.
